# DdCBE-mediated mitochondrial base editing in human 3PN embryos

**DOI:** 10.1038/s41421-021-00358-y

**Published:** 2022-02-01

**Authors:** Xiaoxu Chen, Dong Liang, Jiayin Guo, Junqiang Zhang, Haifeng Sun, Xiaolan Zhang, Jiachuan Jin, Yichen Dai, Qinmin Bao, Xuezhen Qian, Lei Tan, Ping Hu, Xiufeng Ling, Bin Shen, Zhengfeng Xu

**Affiliations:** 1 State Key Laboratory of Reproductive Medicine, Nanjing Medical University, Nanjing, Jiangsu China; 2 Department of Prenatal Diagnosis, Women’s Hospital of Nanjing Medical University, Nanjing Maternity and Child Health Care Hospital, Nanjing, Jiangsu China; 3 Gusu School, Nanjing Medical University, Nanjing, Jiangsu China; 4 Department of Reproduction, Women’s Hospital of Nanjing Medical University, Nanjing Maternity and Child Health Care Hospital, Nanjing, Jiangsu China; 5 Center for Global Health, School of Public Health, Nanjing Medical University, Nanjing, Jiangsu China

**Keywords:** DNA mismatch repair, Genomic analysis

Dear Editor,

The human mitochondrial genome is a double-stranded, circular molecule comprising 16,569 base pairs and contains 37 genes. Mitochondrial dysfunction mediated by mtDNA mutation results in a number of human diseases, often affecting the central nervous and musculoskeletal system^1^. Epidemiology research indicated that the minimum prevalence of mtDNA mutations causing mitochondrial diseases is about 1 in 5000, which is much higher than that of the nuclear gene mutations causing mitochondrial dysfunction^2^. Over 100 pathogenic point mutations have been identified in human mtDNA^3^. In recent years, advances in genome editing technologies have been achieved for precise editing of the nuclear genes, whereas due to the lack of repair mechanism for mtDNA, it is technically challenging to precisely edit mtDNA^4^. In 2020, an mtDNA base editing tool, DddA-derived cytosine base editor (DdCBE), was developed by fusing MTS-TALE to split DddA_tox_ pairs, which could achieve cytosine to thymine (C-to-T) conversion at desired sites in the mtDNA genome of human cell lines^5^. However, the efficacy and specificity of DdCBE still need to be evaluated in in vivo models, especially in human embryos, before pursuing a potential treatment for mtDNA mutation-associated diseases.

Tripronuclear (3PN) zygotes, which are usually discarded in regular clinical pathways, could partially reach the blastocyst stage when being cultured in vitro^6^. In addition, adequate mtDNA still exists in 3PN embryos^7^. Therefore, we set out to use human 3PN embryos as a model system to evaluate the base-editing efficacy and off-target effect of DdCBE during human early embryonic development in this study.

In order to verify the activity of the DdCBE in human cells, three pathogenic mutation sites (G3733A, G8363A, and G13513A), located on human mitochondrial *ND1*, *TRNK*, and *ND5* genes respectively, were chosen for proof-of-principle test in HEK293FT cells. These mutations lead to human mitochondrial disorders such as LHON, MERRF, Leigh Syndrome, and MELAS (Supplementary Table S1). For each site, 4 pairs of DdCBE plasmids with different combinations of orientation and split type (Left-G1333C (L1333C) + Right-G1333N (R1333N), L1397C + R1397N, L1333N + R1333C, L1397N + R1397C) were constructed using the DdCBE assembly kit we developed recently (Supplementary Table S2 and Data S1)^8,9^. HEK293FT cells were transfected with each DdCBE pair and collected on day 6 to amplify the targeted region of mtDNA for Sanger sequencing. We found that DdCBE pairs targeting each of the three sites showed various editing efficiencies in HEK293FT cells (Supplementary Fig. S1a). Besides desired mutations, bystander mutations could also be detected in the spacing region with certain DdCBE pairs, including G3736A induced by L1333C + R1333N pair for G3733, G8361A induced by L1397N + R1397C pair for G8363, C13515T and C13516T induced by L1333N + R1333C pair for G13513 (Supplementary Fig. S1a).

To quantitatively evaluate the editing efficiency of DdCBE pairs, deep sequencing was performed (Supplementary Table S3). The results showed that besides a strong preference for 5′-tC context, C within 5′-aC and 5′-acC motif could also be modified by certain DdCBE pairs, resulting in the occurrence of bystander mutations in the spacing region (Supplementary Fig. S1b‒d). At the G3733 site, L1397C + R1397N and L1397N + R1397C pairs showed higher editing activity than the other two (Supplementary Fig. S1b), while they also converted C ∙ G to T ∙ A at C3738 efficiently within the spacing region, resulting in a silent mutation (both GTC and GTT encode valine). Because L1397C + R1397N also modified C3740 and C3741 sites, the L1397N + R1397C pair was finally selected for human 3PN editing. For G8363 and G13513 sites, L1333C + R1333N and L1397C + R1397N, with high editing performance and fewer bystander mutations, were chosen to edit human 3PN, respectively (Supplementary Fig. S1c, d). Within the spacing region, no indels induced by G3733- and G8363-DdCBEs were detected. Unexpectedly, a single nucleotide deletion at G13513 was detected with very low frequency (< 0.2%) in HEK293FT cells transfected with G13513-DdCBE pairs (Supplementary Fig. S1e). To further test the durability of mtDNA edits in HEK293FT cells, we picked up individual colonies and performed Sanger sequencing. The selected DdCBE pairs could yield almost complete C ∙ G-to-T ∙ A conversion in some clones (Supplementary Fig. S2). Altogether, DdCBE can mediate C ∙ G-to-T ∙ A editing with high frequency at G3733, G8363 and G13513 in HEK293FT cells.

To evaluate the mtDNA editing efficiency of DdCBE in human 3PN embryos, the selected DdCBE pairs were in vitro transcribed to mRNAs by a pre-set T7 promoter and injected into the cytoplasm of human 3PN embryos at concentrations of 100 or 200 ng/μL (Supplementary Table S4). The viable microinjected 3PN embryos were collected on day 3, day 5, and day 6 post injection for Sanger sequencing and whole mtDNA sequencing. It has been reported that mtDNA replication is largely silenced from the fertilized oocyte stage through the preimplantation embryo stage of human^10^. Since DdCBE-mediated base conversion relies on the replication of mtDNA after deamination, inactive mtDNA replication in early embryonic stages would impair the editing outcome. To investigate the status of targeted cytosine in 3PN embryos, we used uracil (U) recognizable DNA polymerase (UC) or U incompatible DNA polymerase (HiFi) to amplify the mtDNA of every single embryo. The result of Sanger sequencing showed that among 3PN embryos injected with G3733-DdCBE mRNAs, embryo No. 20 amplified with UC (#20-UC) and embryo No. 29 amplified with HiFi (#29-HiFi), which were derived from the same donor, were both detected with C ∙ G-to-T ∙ A conversion at G3733, rather than their maternal donor (Fig. 1a). Like in HEK293FT cells, the bystander mutation at C3738 could also be detected by both UC and HiFi polymerases (Fig. 1a). The editing events at the two loci were also detected in other injected 3PN embryos from different donors by both polymerases (Supplementary Fig. S3a). For G8363-DdCBE, C ∙ G-to-T ∙ A conversion at G8363 could be detected by both UC and HiFi polymerases in injected 3PN embryos from different donors (Fig. 1b; Supplementary Fig. S3b). These results demonstrated that the Us induced by DdCBE could be converted to T in 3PN embryos, although mtDNA replication is relatively inactive during early embryonic development. As for G13513, because DdCBE achieved C ∙ G-to-T ∙ A conversion at G13513 with relatively low editing efficiency in embryos (Supplementary Fig. S3c), these embryos were not used for the subsequent analysis. We then performed whole mtDNA sequencing for the 3PN embryos treated with G3733- and G8363-DdCBE. The results showed that the mutation loads of G3733A in edited embryos ranged from 3.67% to 37.54% detected with UC, and from 2.77% to 58.97% detected with HiFi (Fig. 1c; Supplementary Table S5). Except for C3738T, a silent mutation, other bystander mutations within the spacing region were detected with less than 2% editing. Successfully edited embryos for G8363A showed mutation loads ranging from 21.56% to 44.54% detected with UC, and from 1.36% to 10.37% detected with HiFi (Fig. 1d; Supplementary Table S5). The ratio of bystander mutations at C8359 and G8361 was under 3.2%. Taken together, microinjection of selected DdCBE mRNAs can mediate C ∙ G-to-T ∙ A conversion efficiently at desired mtDNA sites in human 3PN embryos with limited bystander mutations.

**Fig. 1:**
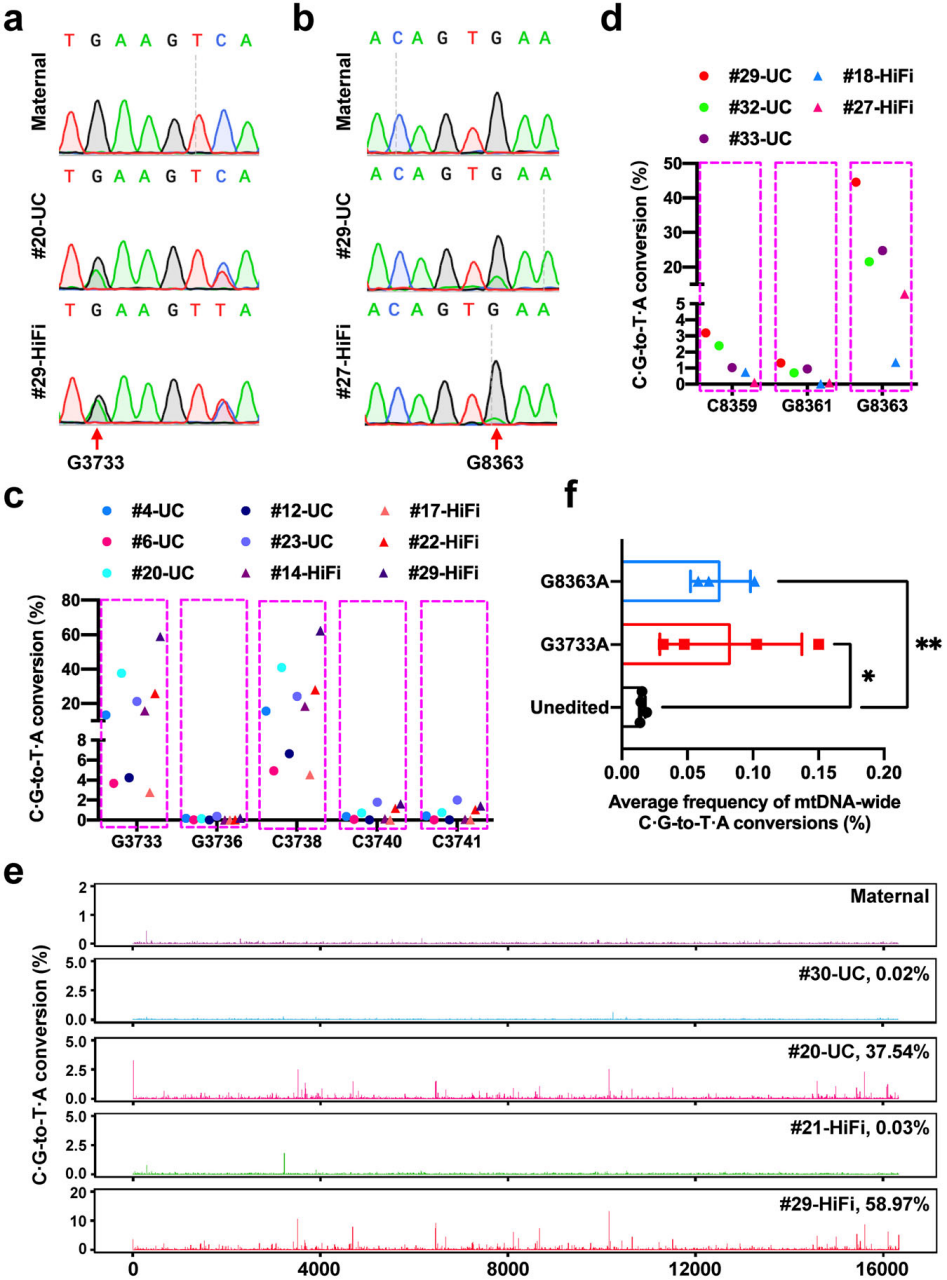
DdCBE-mediated mtDNA base editing in human 3PN embryos. **a** Sequence chromatograms of maternal peripheral blood mononuclear cells (PBMCs) and 3PN embryos injected with G3733-DdCBE mRNAs. #20-UC and #29-HiFi embryos are derived from the same donor. The target site is indicated by the red arrow. **b** Sequence chromatograms of maternal PBMCs and 3PN embryos injected with G8363-DdCBE mRNAs. #29-UC and #27-HiFi embryos are derived from different donors. **c**, **d** Frequencies of C ∙ G-to-T ∙ A conversion within the spacing region of G3733A (**c**) and G8363A (**d**) embryos. Each dot represents an embryo. **e** Frequencies of off-target C ∙ G-to-T ∙ A conversions along the whole mtDNA of maternal PBMC and G3733A embryos. The frequency of on-target editing is labeled behind the embryo number. The four 3PN embryos are from the same donor. **f** Average frequency of mtDNA-wide C ∙ G-to-T ∙ A conversions for each DdCBE pair. Data are presented as means ± SD (*n* = 4 for unedited controls and G3733A embryos, *n* = 3 for G8363A embryos). Significance was calculated with unpaired two-tailed Student’s *t*-test (* P < 0.05, ** P < 0.01).

To profile the off-target activity of DdCBEs on the entire mitochondrial genome, we first performed whole mtDNA sequencing in HEK293FT cells. Off-target events could be detected along the mitochondrial genome in cells transfected with DdCBE, rather than Dead ND1-DdCBE (Supplementary Fig. S4a and Table S3). G3733-, G8363-, and G13513-DdCBE yielded the highest off-target editing efficiencies of 4.92% at C10205, 6.78% at G8115, 6.76% at C15598, respectively (Supplementary Fig. S4a and Table S3). The average frequencies of mitochondrial genome-wide off-target editing induced by the three DdCBEs were significantly higher than that of control cells (Supplementary Fig. S4b). The off-target sites (OTS) with an average conversion rate of over 1% were selected for further analysis (37 sites for G3733-DdCBE, 94 sites for G8363-DdCBE, and 51 sites for G13513-DdCBE) (Supplementary Fig. S4c). All the OTS were detected within 5′-tC motifs, suggesting that the off-target editing was indeed caused by DddA_tox_ (Supplementary Table S6). Among these OTS, 16 sites were shared by the three DdCBE pairs, and showed higher average off-target editing efficiency as compared with unmerged OTS (Supplementary Fig. S4d, e). Notably, 6 out of these 16 OTS were also identified in a previous study (Supplementary Fig. S4f)^5^. Moreover, in the case of allowing 4-base mismatches and limiting OTS 20 bp upstream or downstream of the TALE array recognition site, only 2 sites (2/133) could be detected which might be induced by the sequence-dependent activity of DdCBE (Supplementary Fig. S5). These results suggest that the OTS are mostly caused by the sequence-independent activity of DdCBE.

To examine the off-target effect of the DdCBEs in human 3PN embryos, we analyzed the mitochondrial genome-wide C ∙ G-to-T ∙ A conversions from the result of whole mtDNA sequencing and found that off-target events were detected along the mitochondrial genome by both UC and HiFi polymerases in edited embryos (Fig. 1e; Supplementary S6a, b and Table S5). For instance, among G3733A #20-UC, #21-HiFi, #29-HiFi, and #30-UC embryos, which were all derived from the same maternal donor, #20-UC and #29-HiFi could be detected with 14 and 116 OTS (conversion rate over 1%) respectively, whereas these OTS were hardly detected in the unedited embryos (#21-HiFi and #30-UC) and their maternal donor (Fig. 1e). In addition, embryos harboring higher on-target editing, also showed more OTS (Supplementary Fig. S6a, b), indicating a strong correlation between on-target editing efficiency and off-target activity of DdCBE. For example, G3733A #22-HiFi embryo harboring 25.82% on-target editing had 39 OTS, whereas G3733A #14-HiFi embryo with 15.68% on-target editing had no OTS; G8363A #29-UC embryo (44.54%) had 2.58-fold more OTS compared with G8363 #32-UC (21.56%) embryo (Supplementary Fig. S6a, b). To characterize more authentic OTS, embryos with over 20% on-target editing were selected for further study. The average frequencies of mitochondrial genome-wide off-target editing of these well-edited embryos were significantly higher than that of unedited embryos (Fig. 1f). 255 and 137 OTS with over 1% editing were identified in G3733A and G8363A embryos respectively (Supplementary Fig. S7a). Among these OTS, 96.47% and 97.81% OTS were detected within 5′-tC motif (Supplementary Fig. S7b), and 106 OTS could be detected in both G3733A and G8363A embryos (Supplementary Fig. S7a), suggesting that the off-target editing was mostly caused by DddA_tox_. 100% (37/37) and 88.30% (83/94) OTS detected in HEK293FT cells were reproduced in G3733A and G8363A embryos, respectively (Supplementary Fig. S7c, d). Taken together, the DdCBE pair has a noticeable off-target activity in human 3PN embryos.

In summary, our work demonstrated the feasibility of DdCBE-mediated mitochondrial base editing in human 3PN embryos for the first time, suggesting a possibility of pathogenic mtDNA mutation correction in the human early embryonic stage. A series of pathogenic A ∙ T-to-G ∙ C mutations in mtDNA, including A3260G, A4300G, T7510C, T7511C, T8356C, and A14495G, could be theoretically corrected by DdCBE to achieve a therapeutic result. Although the off-target editing detected in 3PN mtDNA may not be sufficient to result in phenotypes, the current mitochondrial base editing strategy requires further optimization to meet the demand of any clinical application.

## Supplementary information

Supplementary information

Supplementary table S3

Supplementary table S5

Supplementary table S8

## Data Availability

The high-throughput sequencing data have been deposited to the NCBI Sequence Read Archive database under the accession ID PRJNA758504.
