# Supplementary material for: DdCBE-mediated mitochondrial base editing in human 3PN embryos
Source: Cell Discov. 2022 Feb 1;8:8. doi: 10.1038/s41421-021-00358-y (PMC8803914; doi:10.1038/s41421-021-00358-y)
Supplement: Supplementary file 1 — Supplementary information [file 41421_2021_358_MOESM1_ESM.pdf]

Supplementary information

DdCBE-mediated mitochondrial base editing in human 3PN embryos

Contents

Materials and Methods

Figure S1. DdCBE-mediated mtDNA editing in HEK293FT cells.

Figure S2. Sanger sequencing result of single clone of HEK293FT cells treated with G3733-, G8363- and G13513-DdCBE pairs.

Figure S3. Sanger sequencing result of 3PN embryo.

Figure S4. DdCBE-mediated off-target editing in HEK293FT cells.

Figure S5. Putative off-target sites induced by the sequence-dependent activity of DdCBE.

Figure S6. DdCBE-mediated off-target editing in whole mtDNA of human 3PN embryos.

Figure S7. Characteristics of DdCBE-mediated off-target editing in human 3PN embryos.

Table S1. mtDNA mutations and mitochondrial diseases.

Table S2. TALE array binding sequence.

Table S3. Whole mtDNA sequencing results of HEK293FT cells (excel file).

Table S4. Summary of mtDNA base editing efficiency in human 3PN embryos.

Table S5. Whole mtDNA sequencing results of human 3PN embryos (excel file).

Table S6. Summary of off-target motif in HEK293FT cells.

Table S7. Primers information.

Table S8. List of excluded SNPs (excel file).

Supplementary Data S1. Plasmid information of this study.

Materials and Methods

DdCBE plasmids construction

All DdCBE vectors were assembled using RVD libraries as described before [1, 2], which can be obtained from Addgene (Shen Lab DdCBE kit, pending). In brief, backbone and RVDs plasmids were digested with Bsa I (New England Biolabs) and ligated with T4 DNA ligase (New England Biolabs) in a single tube using the following program: 37°C for 10 min; 10 cycles of 10 min at 37°C and 10 min at 16°C; 50°C for 5 min; 80°C for 5 min. The assembled plasmids were chemically transformed into *Escherichia coli* DH5 α competent cells (Transgene), and then confirmed by PCR and Sanger sequencing. PCR primers and sequencing primers are listed in Supplementary Table S7. Sequences of DdCBE plasmids constructed in this study are list in Supplementary Data S1.

Cell culture and nucleofection

HEK293FT cells (Thermofisher, R70007) were maintained in DMEM with 10% FBS (Germini) at 37°C with 5% CO_2, and detected without mycoplasma contamination by PCR test. Cells (200,000) were co-transfected with 400 ng of left and right DdCBE each using SF Cell Line 4D-Nucleofector X Kit (Lonza) according to the manufacturer's protocol. The nucleofected cells were treated with 2 μg/mL puromycin 24 hrs post nucleofection, and collected at day 6 for further analysis.

DNA extraction and genotyping

Genomic DNA of HEK293FT cell were extracted with QuickExtract™ DNA Extraction Solution (Lucigen) according to the manufacturer's protocol. The editing

regions of each site were amplified for Sanger sequencing. Primers are listed in Supplementary Table S7.

In vitro transcription of DdCBE

DdCBE pair was linearized with Pme I (New England Biolabs) and transcribed using mMESSAGE mMACHINE T7 Ultra Kit (Life Technologies) according to the manufacturer's manual. The transcribed mRNA was purified by RNA Clean & Concentrator™-25 (Zymo Research) and stored at -80°C until use.

Identification and collection of human tripronulear (3PN) zygotes

Cumulus-Oocyte Complexes (COCs) were picked up from follicular fluid, washed in culture medium G-IVF (Vitrolife, Gothenburg, Sweden), and cultured in the tri-gas incubator (APM-30D, ASTEC, Tokyo, Japan) at 37°C with 6% CO_2, 5% O_2 and 89% N_2. Subsequently, the oocytes were inseminated by in vitro fertilization (IVF) or intracytoplasmic sperm injection (ICSI) as previously described [3]. After 16-18 hrs, the zygotes with two clear pronuclei were identified to confirm normal fertilization, while the abnormal zygotes with three pronuclei were selected and cryopreserved for further usage in this study. From August 2020 to December 2020, a total of 112 3PN human zygotes were obtained from Nanjing Maternity and Child Health Care Hospital. This study conformed to ethical standards of Helsinki Declaration and national legislation and was approved by the Institutional Review Board (IRB) of the Nanjing Maternity and Child Health Care Hospital (2020 KY-063, August 27th 2020).

Vitrification and recovery of the 3PN zygotes

3PN zygotes were frozen and thawed in the same method as the procedure with cleavage stage embryos and blastocysts. Vitrification and warming solutions (Kitazato BioPharma Co., Shizuoka, Japan) were prepared in advance at room temperature. For vitrification, 3PN zygotes were firstly placed in solution ES containing 7.5% ethylene glycol (EG) and 7.5% dimethylsulfoxide (DMSO) for 10-12 min. After shrinkage recovery, they were transferred into solution VS containing 15% EG, 15% DMSO and 0.5 M sucrose for 1 min. Finally, each 3PN zygote was put on the film strip of the Cryotop device and submerged into liquid nitrogen quickly. For thawing, 3PN zygotes on Cryotop device were placed in thawing solution containing 1.0 M sucrose for 1 min, then transferred into thawing solution DS containing 0.5 M sucrose for 3 min. After that, 3PN zygotes were incubated successively in thawing solution WS1 and WS2 for 5 min each, and the 3PN zygotes were finally transferred into culture medium G2 (Vitrolife, Gothenburg, Sweden) for subsequent analysis.

3PN zygotes injection and culture

The thawed 3PN zygotes were placed in the injection dish. DdCBE mRNA was diluted into working concentration using RNase free water before injection. Microinjection was performed under an inverted microscope (Nikon, Japan) equipped with micromanipulators and microinjector (Eppendorf, Germany). The injected 3PN zygotes were then transferred into pre-equilibrated culture medium G1 (Vitrolife, Gothenburg, Sweden) and cultured for 2 days. Afterwards, the 3PN embryos were transferred into culture medium G2 (Vitrolife, Gothenburg, Sweden) until the embryos reached the blastocyst stage (Day 5–6). The blastocysts were scored according to Gardner's

standard. During this period, the morphologies of 3PN embryos, including cell number, fragmentation and regularity of the blastomeres, were daily checked. The embryos were collected for Tn5 tagmentation when they met either criteria: embryos with obvious developmental delay or embryos reached the blastocyst stage.

Mito-ATAC-seq and genotyping of 3PN embryos

Mito-ATAC-seq was performed for whole mitochondrial genome sequencing of single 3PN embryo as described before with minor modifications [4]. Briefly, single human 3PN embryo was tagmented at 37°C for 30 min in a thermomixer with 0.5 μL of Tn5 transposase (Vazyme, TD501) in a total volume of 10 μL buffer containing 1X TD buffer, 0.1% NP-40 and 0.3X PBS. Then tagmented fragments were amplified with barcoded primers (Vazyme, TD204-207) using KAPA HiFi HotStart ReadyMix (Roche, KK2602) or Phanta UC Super-Fidelity DNA Polymerase (Vazyme, P507-01). 1 μL of the unpurified library was used as templates to amplify the editing region for genotyping by Sanger sequencing. The library of 200–1000 bp was recovered with the FastPure Gel DNA Extraction Mini Kit (Vazyme, DC301-01) and sequenced on Illumina Novaseq platform.

Deep sequencing

The editing efficiency of on-target site was calculated by deep sequencing in HEK293FT cells. Briefly, first round PCR (PCR1) was used to amplify the editing regions with barcoded primer using Phanta Max Super-Fidelity DNA Polymerase (Vazyme, P505) for 25 cycles. And then equal molar PCR1 products were pooled for the second round PCR (PCR2) with VAHTS™ Multiplex Oligos set 4 for Illumina

(Vazyme, N321) using KAPA HiFi HotStart ReadyMix for 6 cycles. The second round PCR (PCR2) products were purified using DNA Clean Beads (Vazyme, N411) and sequenced on Illumina Novaseq platform. Primers for PCR1 are listed in Supplementary Table S7.

Whole mitochondrial DNA sequencing

Maternal mitochondrial DNA of peripheral blood cells was captured by long-range PCR of two overlapping mitochondrial fragments with Phanta Max Super-Fidelity DNA Polymerase and recovered by gel extraction. The two fragments were pooled with equal moles and subjected to library preparation using TruePrepTM DNA Library Prep Kit V2 for Illumina (Vazyme, TD501). The library was purified using DNA Clean Beads by 0.5X / 0.35X double size selection and sequenced on Illumina Novaseq platform. Primers for amplification of mitochondrial fragment DNA are listed in Supplementary Table S7.

Tn5 transposase was used to capture the mtDNA of HEK293FT cells as previously described with modifications [5]. Briefly, 10^5 cells were lysated with 20 μL of cold RSB containing 0.1% NP40, 0.1% Tween-20, and 0.01% Digitonin. The nuclei were pelleted at 500 RCF for 10 min at 4°C. 9.5 μL of supernatant containing mitochondria was collected and added with 0.5 μL of Tn5 transposase and 10 μL of 2X TD buffer. The reaction was incubated at 37°C for 30 min in a thermomixer with 1,000 rpm mixing. The transposed fragments were amplified using TruePrep DNA Library Prep Kit V2 (Vazyme, TD501). The library was purified by DNA Clean Beads by 0.5X / 0.35X double size selection and sequenced on Illumina Novaseq platform.

Deep sequencing data analysis

The human mitochondrial genome reference sequence (NC_012920.1) was downloaded from NCBI database. The quality control of sequencing data was performed by fastqc and trim_galore in paired end mode. Bowtie2 was used to build the alignment index using default parameters. Paired end reads with overlap were merged into a single read, and bowtie2 was used for alignment in single end mode. Otherwise, reads were mapped in paired end mode by using bowtie2 with default parameters. Alignment results were converted to bam format using samtools and visualized in Integrative Genomics Viewer (IGV). Bases with depth over 2 million were truncated to 2 million, and only C-to-T or G-to-A conversion was calculated for DdCBE-mediated editing. To analyze the indels, VanScan2 was used as previously described [4].

Whole mtDNA sequencing data analysis

The quality control of sequencing data was performed by fastqc and trim_galore in paired end mode. The Illumina adapter sequence or Ns in either side of the read was trimmed, and only reads with quality over 20 were kept for further analysis. QC-passed reads were mapped to NC_012920.1 by using bowtie2 with default parameters of paired end. The DdCBE editing efficiency was calculated as mentioned above.

Off-target analysis

For the off-target analysis in 3PN embryos, the following sites were excluded before analysis and visualization: (1) SNP sites with C·G to T·A variation of East Asian were obtained from the NCBI database; (2) the sites with C·G to T·A variation over 1% in

any maternal sample; (3) the evident SNP sites with C·G to T·A variation over 90% in any 3PN sample; (4) sites within the DdCBE spacing region. Excluded SNPs are listed in Supplementary Table S8. For the off-target analysis in HEK293FT cells, sites with C·G-to-T·A variation over 1% in control cells were excluded. The average off-target editing frequency for each sample was calculated as: the sum editing frequencies of all sites with C·G-to-T·A conversion were divided by the total number of sites with C·G-to-T·A conversion.

Ethics approval

This study conformed to ethical standards of Helsinki Declaration and national legislation and was approved by the Institutional Review Board (IRB) of the Nanjing Maternity and Child Health Care Hospital (2020 KY-063, August 27th 2020).

Consent to participate

Informed consent was obtained from all the donors of the 3PN zygotes in this study.

Reference

1 Guo, J. *et al*. Precision modeling of mitochondrial disease in rats via DdCBE-mediated mtDNA editing. *Cell Discov* **7**, 78 (2021).

2 Qi, X. *et al*. Precision modeling of mitochondrial diseases in zebrafish via DdCBE-mediated mtDNA base editing. *Cell Discov*. 10.1038/s41421-021-00325-7 (2021).

3 Chen, X. *et al.* Trophectoderm morphology predicts outcomes of pregnancy in vitrified-warmed single-blastocyst transfer cycle in a Chinese population. *Journal of assisted reproduction and genetics* **31**, 1475-1481 (2014).

4 Mok, B. Y. *et al.* A bacterial cytidine deaminase toxin enables CRISPR-free mitochondrial base editing. *Nature* **583**, 631-637 (2020).

5 Corces, M. R. *et al.* An improved ATAC-seq protocol reduces background and enables interrogation of frozen tissues. *Nature methods* **14**, 959-962 (2017).

Figure S1

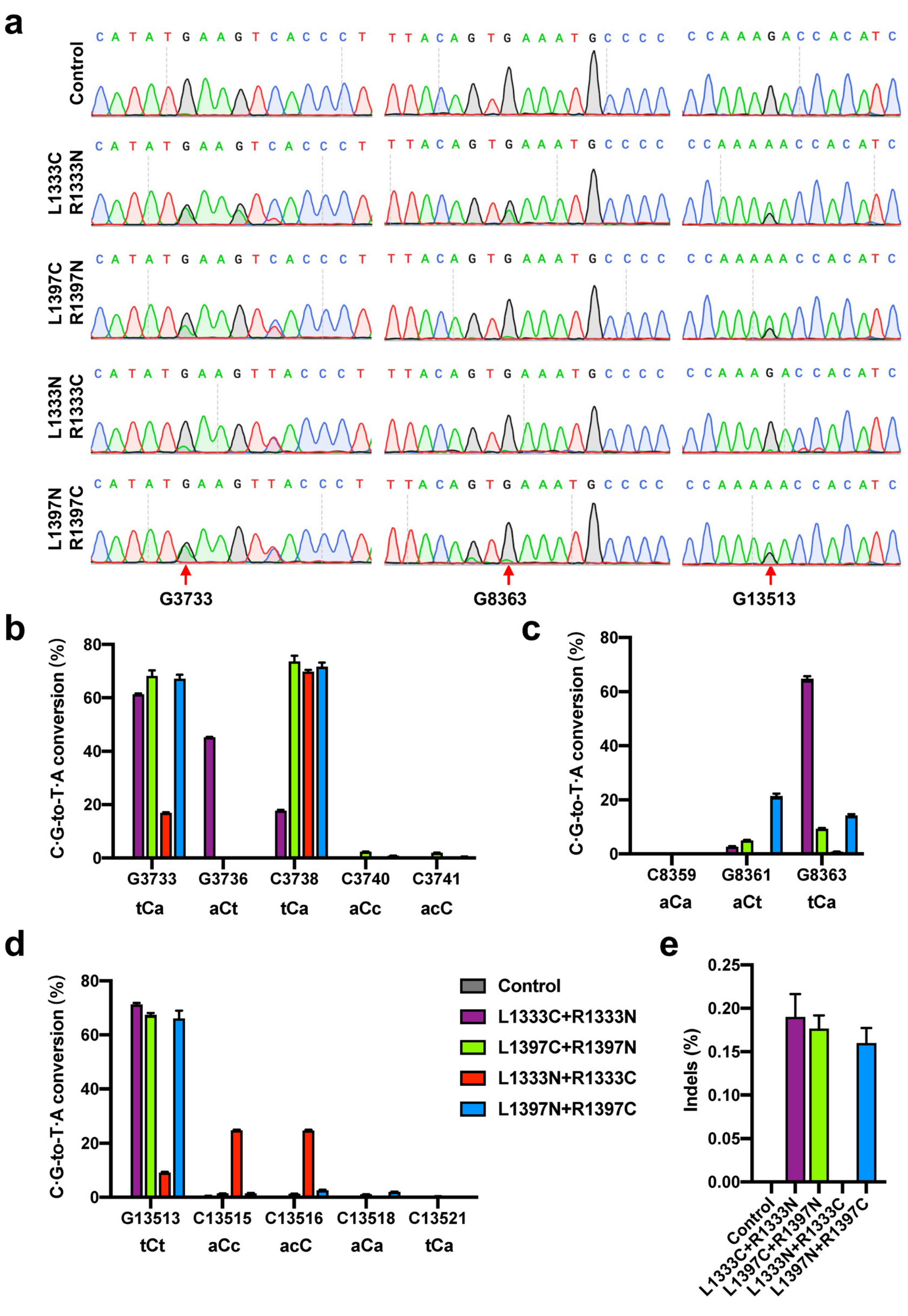

Figure S1. DdCBE-mediated mtDNA editing in HEK293FT cells.

(a) Sequence chromatograms of HEK293FT cells treated with G3733-, G8363- and G13513-DdCBE pairs. Untreated cells were used as control. Targeted sites are indicated with red arrows.

(b-d) Frequencies of C·G-to-T·A conversion within spacing region of HEK293FT cells treated with G3733-, G8363- and G13513-DdCBE pairs. Data are presented as means ± SD (*n* = 1 for untreated control, *n* = 3 for each DdCBE pair). The motifs are listed below each edited site.

(e) Indels frequencies within spacing region of HEK293FT cells treated with G13513-DdCBE pairs. Untreated cells were used as control. Data are presented as means ± SD (*n* = 1 for untreated control, *n* = 3 for each DdCBE pair).

Figure S2

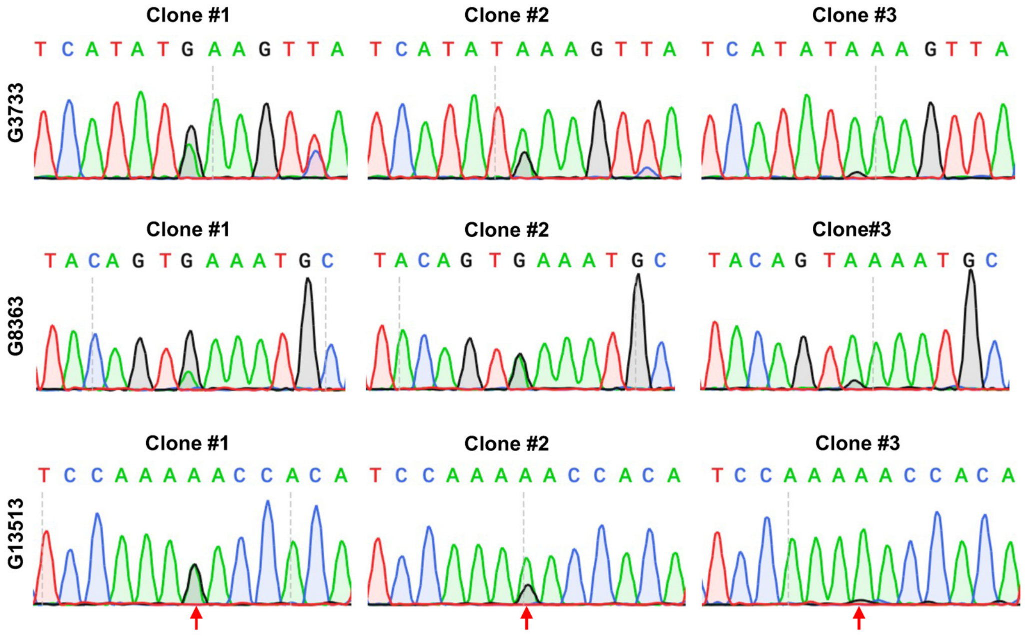

Figure S2. Sanger sequencing result of single clone of HEK293FT cells treated with G3733-, G8363- and G13513-DdCBE pairs.

Representative sequence chromatograms of single clone with low, moderate and high mutation loads.

Figure S3

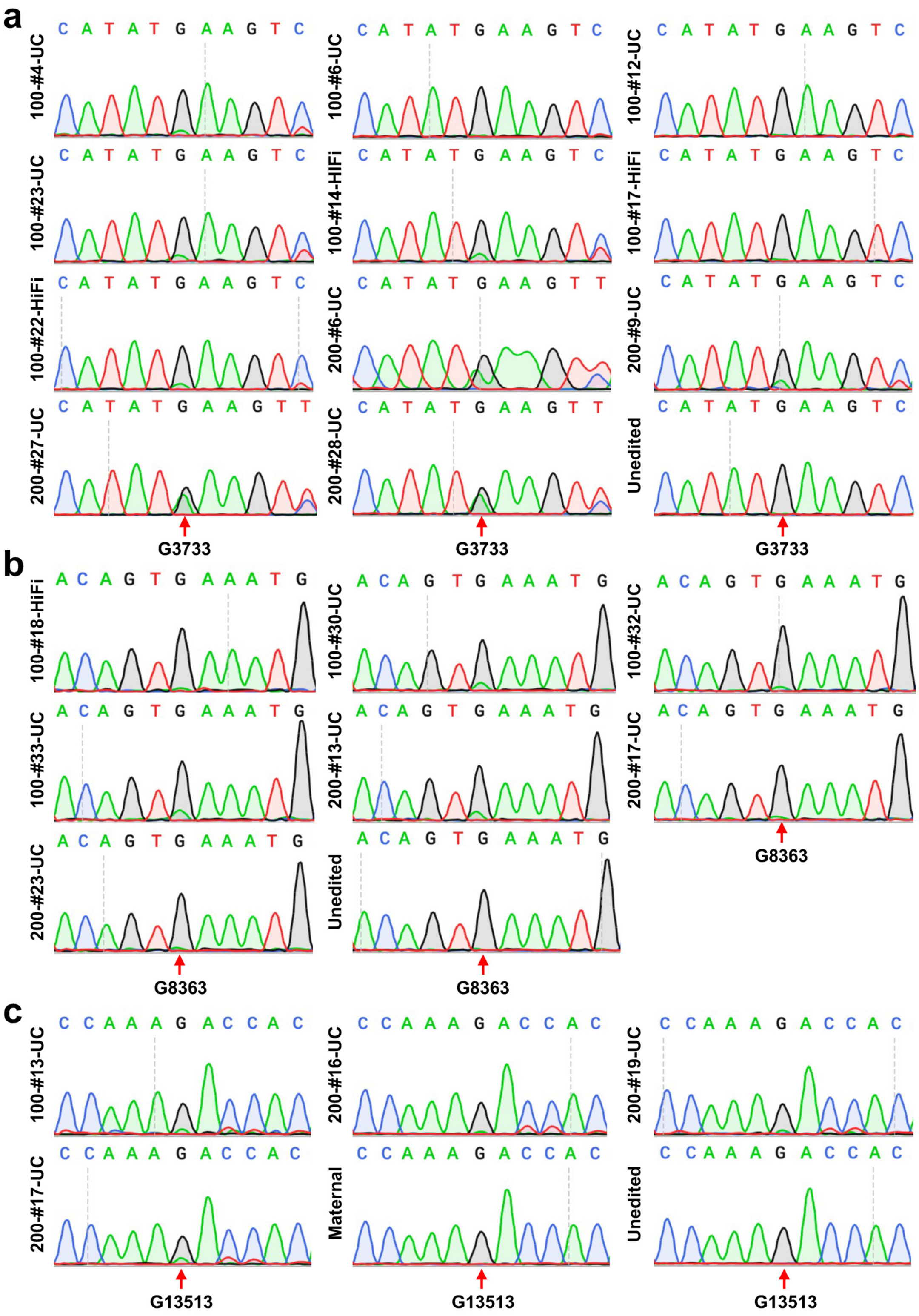

Figure S3. Sanger sequencing result of 3PN embryo.

(a-c) Sequence chromatograms of donors and 3PN embryos injected with G3733- (a), G8363- (b) and G13513-DdCBE (c) mRNAs at concentrations of 100 and 200 ng/μL. Target sites are indicated by red arrows.

Figure S4

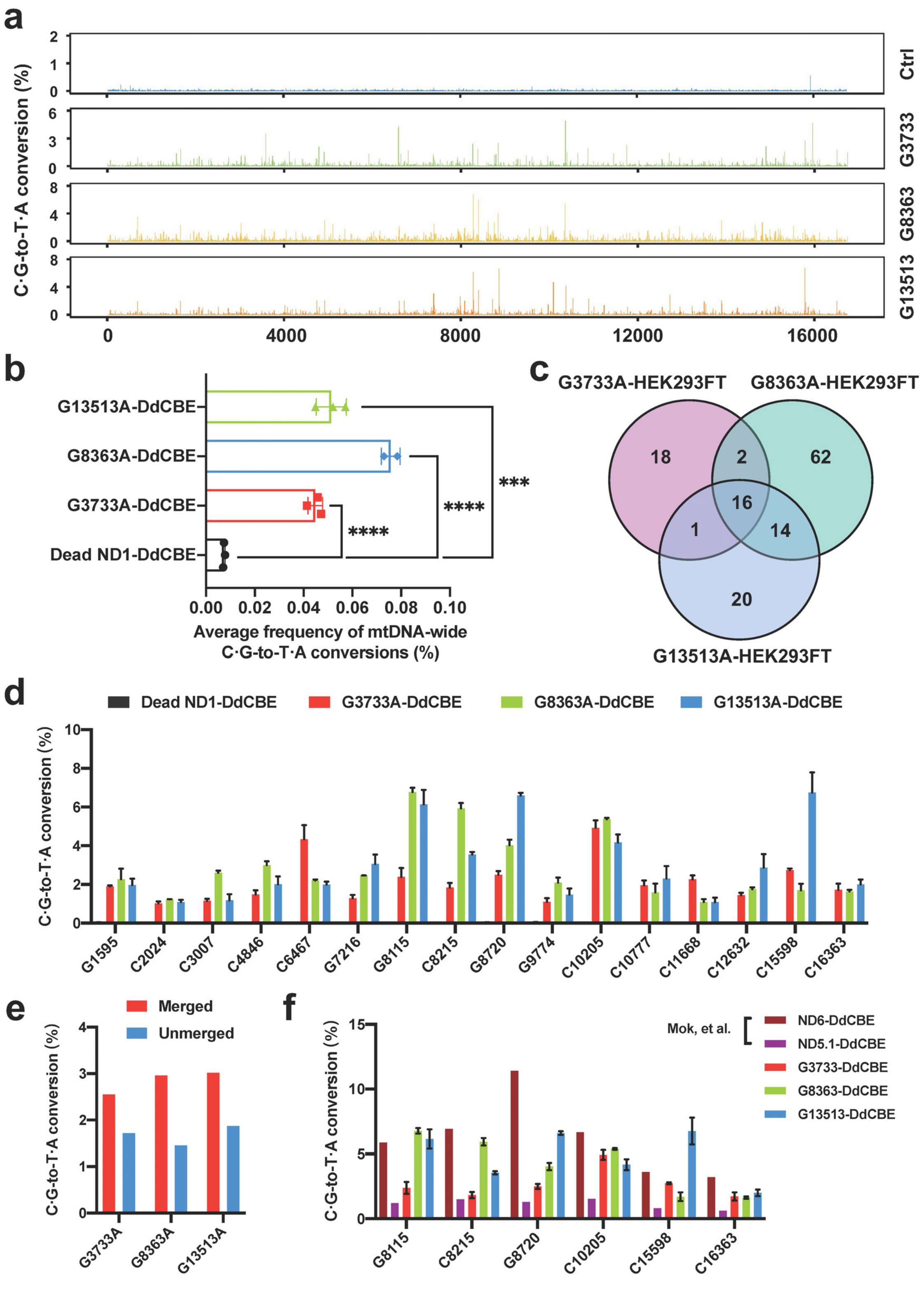

Figure S4. DdCBE-mediated off-target editing in HEK293FT cells.

(a) Frequencies of C·G-to-T·A conversions along the whole mtDNA of HEK293FT cells treated with G3733-, G8363- and G13513- DdCBE pairs. Dead ND1-DdCBE pair was used as control.

(b) Average frequency of mtDNA-wide C·G-to-T·A conversions for each DdCBE pair. Data are presented as means ± SD (n = 3 for Dead ND1-, G3733- and G13513-DdCBE, n = 2 for G8363-DdCBE). Significance was calculated with unpaired two-tailed Student's t-test (*** $P < 0.001$, **** $P < 0.0001$).

(c) The overlapped off-target sites in G3733A, G8363A and G13513A HEK293FT cells.

(d) Editing efficiencies of 16 overlapped off-target sites in G3733A, G8363A and G13513A HEK293FT cells. Data are presented as means ± SD (n = 3 for Dead ND1-, G3733- and G13513- DdCBE, n = 2 for G8363-DdCBE).

(e) Average frequency of merged and unmerged off-target sites in G3733A, G8363A and G13513A HEK293FT cells.

(f) Editing efficiencies of overlapped off-target sites between this study and a previous report.

Figure S5

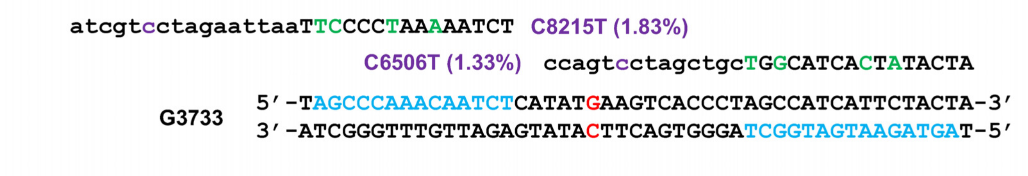

Figure S5. Putative off-target sites induced by the sequence-dependent activity of DdCBE.

The target base is shown in red. RVD array recognition sequences are in blue. Mismatched bases are in green. Off-target sites are in purple.

Figure S6

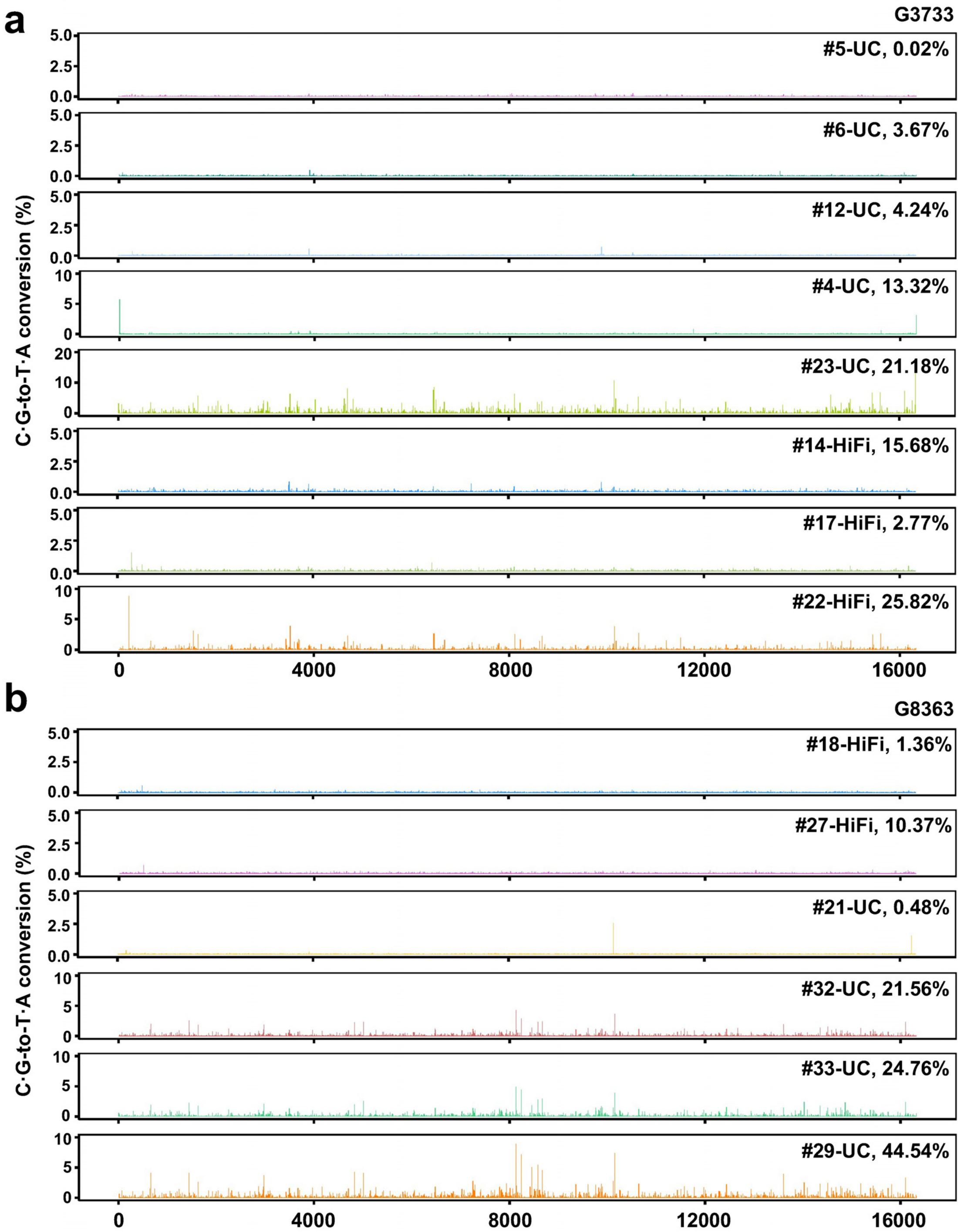

Figure S6. DdCBE-mediated off-target editing in whole mtDNA of human 3PN embryos.

(a, b) Frequencies of off-target C·G-to-T·A conversions along the whole mtDNA of G3733A (a) and G8363A (b) embryos. Frequency of on-target editing is labeled behind the embryo number.

Figure S7

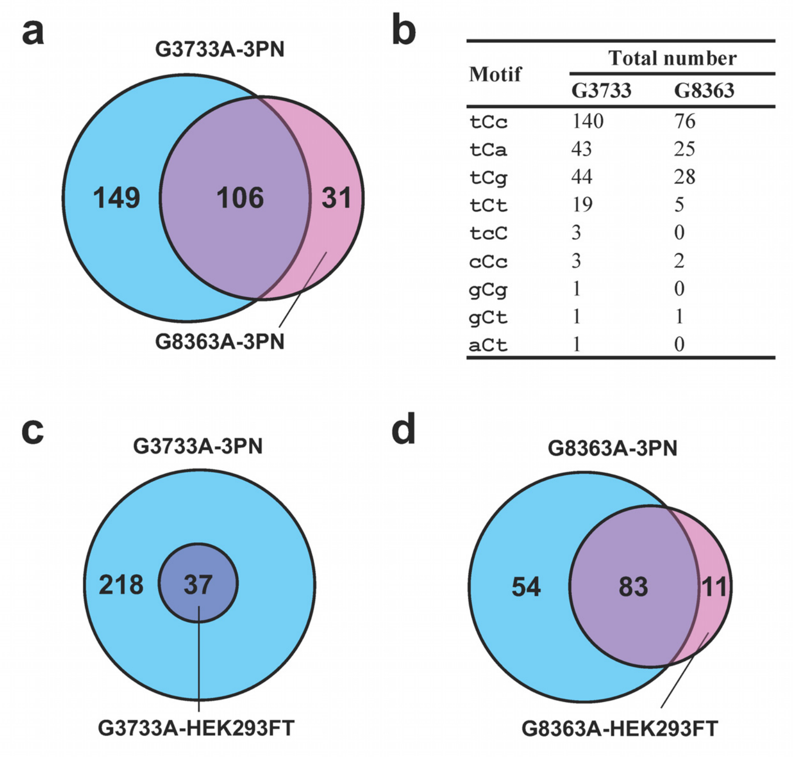

Motif	Total number	
	G3733	G8363
tCc	140	76
tCa	43	25
tCg	44	28
tCt	19	5
tcC	3	0
cCc	3	2
gCg	1	0
gCt	1	1
aCt	1	0

Figure S7. Characteristics of DdCBE-mediated off-target editing in human 3PN embryos.

(a) The overlapped off-target sites between G3733A and G8363A embryos.

(b) Summary of off-target motif in G3733A and G8363A embryos.

(c) The overlapped off-target sites between G3733A 3PN embryos and G3733A HEK293FT cells.

(d) The overlapped off-target sites between G8363A 3PN embryos and G8363A HEK293FT cells.

Table S1. mtDNA mutations and mitochondrial diseases.

Mutation	Location	Syndrome
G3733A	*MT-ND1*	Leber Hereditary Optic Neuropathy (LHON)
G8363A	*MT-TK*	Maternally Inherited Cardiomyopathy (MICM)+Deaf / Myoclonus Epilepsy with Ragged-red Fibers (MERRF) / Autism / Leigh Syndrome (LS) / Ataxia + Lipomas
G13513A	*MT-ND5*	Leigh Disease / Mitochondrial Encephalopathy, Lactic Acidosis and Stroke-like Episodes (MELAS) / LHON-MELAS Overlap Syndrome

Table S2. TALE array binding sequence.

Site	TALE array binding sequence
**G3733**	5′-TAGCCCAAACAATCTCATATGAAGTCACCCTAGCCATCATTCTACTA-3′<br>3′-ATCGGGTTTGTTAGAGTATACTTCAGTGGGATCGGTAGTAAGATGAT-5′
**G8363**	5′-TAAGAGAACCAACACCTCTTTACAGTGAAATGCCCCAACTAAATACTACCGTA-3′<br>3′-ATTCTCTTGGTTGTGGAGAAATGTCACTTTACGGGGTTGATTTATGATGGCAT-5′
**G13513**	5′-TCCTCACAGGTTTCTACTCCAAAGACCACATCATCGAAACCGCAAACA-3′<br>3′-AGGAGTGTCCAAAGATGAGGTTTCTGGTGTAGTAGCTTTGGCGTTTGT-5′

The target base is shown in red. RVD array recognition sequences are in blue.

Table S4. Summary of mtDNA base editing efficiency in human 3PN embryos.

Target Sites	Concentration (ng/μL)	No. of injected 3PN embryo	No. of edited 3PN embryo	Editing ratio	Mutation load
G3733	100	30	9	30%	2.77-58.97%
	200	12	4	33.33%	N.A.
G8363	100	30	6	20%	1.36-44.54%
	200	17	3	17.65%	N.A.
G13513	100	10	1	10%	N.A.
	200	13	3	23.08%	N.A.

Editing ratio is calculated as No. of edited 3PN embryos / No. of injected 3PN embryos. Mutation load is calculated as reads with intended C·G-to-T·A conversion / total reads. Embryos which were not selected to perform the whole mtDNA sequencing to evaluate the mutation loads were labeled with N.A. (not available). Out of the total 112 3NP embryos, only 8 developed to blastocyst stage (7.14%), and none of them were detected with obvious C·G-to-T·A conversion by Sanger sequencing.

Table S6. Summary of off-target motif in HEK293FT cells.

Motif	Total number		
	G3733	G8363	G13513
**tCc**	26	57	38
**tCa**	3	14	4
**tCg**	6	20	7
**tCt**	2	3	2

Table S7. Primers information.

	Primers	Sequence (5'-3')
**Primers for RVD**	RVD seq Fwd	TGACCGCAGTGGAGGCAGTG
	RVD seq Rev	TTCACTGCATCCAGCGCAGG
**Primers for genotyping**	homo-G3733-Fwd	ATGGCCAACCTCCTACTCCT
	homo-G3733-Rev	AGATTGTAGTGGTGAGGGTGTTT
	homo -G8363-Fwd	TGGGTGGTTGGTGTAAATGA
	homo -G8363-Rev	ATCTGTGGAGCAAACCACAGTTTC
	homo-G13513-Fwd	AACTGCTAACTCATGCCCCC
	homo-G13513-Rev	TAGTAAGGGTGGGGAAGCGA
**Barcoded Primers for PCR1**	i5-homo-G3733-Fwd1	ACACTCTTTCCCTACACGACGCTCTTCCGATCTCAAA CCTCTAGCCTAGCCGTTTACT
	i5-homo-G3733-Fwd2	ACACTCTTTCCCTACACGACGCTCTTCCGATCTTCAA CCTCTAGCCTAGCCGTTTACT
	i5-homo-G3733-Fwd3	ACACTCTTTCCCTACACGACGCTCTTCCGATCTACCA CCTCTAGCCTAGCCGTTTACT
	i7-homo-G3733-Rev	GTGACTGGAGTTCAGACGTGTGCTCTTCCGATCTGG ACTCCCCTTCGGCAAG
	i5-homo-G8363-Fwd1	ACACTCTTTCCCTACACGACGCTCTTCCGATCTCAAG CAAACCACAGTTTCATGCCCA
	i5-homo-G8363-Fwd2	ACACTCTTTCCCTACACGACGCTCTTCCGATCTTCAG CAAACCACAGTTTCATGCCCA
	i5-homo-G8363-Fwd3	ACACTCTTTCCCTACACGACGCTCTTCCGATCTACCG CAAACCACAGTTTCATGCCCA
	i7-homo-G8363-Rev	GTGACTGGAGTTCAGACGTGTGCTCTTCCGATCTTT ATGGTGGGCCATACGGTAGTA
	i5-homo-G13513-Fwd1	ACACTCTTTCCCTACACGACGCTCTTCCGATCTCAAA CCATTGGCAGCCTAGCATT
	i5-homo-G13513-Fwd2	ACACTCTTTCCCTACACGACGCTCTTCCGATCTTCAA CCATTGGCAGCCTAGCATT
	i5-homo-G13513-Fwd3	ACACTCTTTCCCTACACGACGCTCTTCCGATCTACCA CCATTGGCAGCCTAGCATT
	i7-homo-G13513-Rev	GTGACTGGAGTTCAGACGTGTGCTCTTCCGATCTGT TGTTTGGAAGGGGGATGC
**Primers for long-range PCR**	homo-mtDNA-F1-Fwd	GCAAATCTTACCCCGCCTG
	homo-mtDNA-F1-Rev	AATTAGGCTGTGGGTGGTTG
	homo-mtDNA-F2-Fwd	GCCATACTAGTCTTTGCCGC
	homo-mtDNA-F2-Rev	GGCAGGTCAATTTCACTGG

Supplementary Data S1. Plasmid information of this study.

Amino acid sequences of Dead ND1- and G3733-DdCBE are annotated as: red for MTS, italics for linker, yellow for flag tag, green for N&C-terminal domain, underlined for RVDs, purple for split $DddA_{tox}$ halves, cyan for UGI. Only RVDs sequences are showed for G8363- and G13513- DdCBE. The red font means the mutation site of E1347A.

Human Dead ND1 Left TALE-G1333C (E1347A)

MLGFVGRVAAAPASGALRRLTPSASLPPAQLLLRAAPTAVHPVRDYAAQTSESGGGG
SPG*AAA*DYKDDDDK*GS*VDLRTLGYSQQQQEKIKPKVRSTVAQHHEALVGHGFTHAHI
VALSQHPAALGTVAVKYQDMIAALPEATHEAIVGVGKQWSGARALEALLTVAGELRG
PPLQLDTGQLLKIAKRGGVTAVEAVHAWRNALTGAPLNLTPEQVVAIASHDGGKQAL
ETVQRLLPVLCQAHGLTPAQVVAIASNGGGKQALETVQRLLPVLCQAHGLTPEQVVA
IASNIGGKQALETVQRLLPVLCQAHGLTPDQVVAIASNNGGKQALETVQRLLPVLCQ
AHGLTPEQVVAIASHDGGKQALETVQRLLPVLCQAHGLTPAQVVAIASHDGGKQALE
TVQRLLPVLCQAHGLTPAQVVAIASNGGGKQALETVQRLLPVLCQAHGLTPEQVVAI
ASNIGGKQALETVQRLLPVLCQAHGLTPDQVVAIASNNGGKQALETVQRLLPVLCQA
HGLTPEQVVAIASHDGGKQALETVQRLLPVLCQAHGLTPAQVVAIASHDGGKQALET
VQRLLPVLCQAHGLTPAQVVAIASNNGGKQALETVQRLLPVLCQAHGLTPEQVVAIA
SNGGGKQALETVQRLLPVLCQAHGLTPEQVVAIASNGGGKQALETVQRLLPVLCQAH
GLTPEQVVAIASNGGGRPALESIVAQLSRPDPALAALTNDHLVALACLGGRPALDAV
KKGLG*GS*PTPYPNYANAGHVAGQSALFMRDNGISEGLVFHNNPEGTCGFCVNMTETL
LPENAKMTVVPPEGAIPVKRGATGETKVFTGNSNSPKSPTKGGC*SGGS*TNLSDIIEK
ETGKQLVIQESILMLPEEVEEVIGNKPESDILVHTAYDESTDENVMLLTSDAPEYKP
WALVIQDSNGENKIKML

Human Dead ND1 Right TALE-G1333N

MLGFVGRVAAAPASGALRRLTPSASLPPAQLLLRAAPTAVHPVRDYAAQTSESGGGG
SPG*AAA*DYKDDDDK*GS*VDLRTLGYSQQQQEKIKPKVRSTVAQHHEALVGHGFTHAHI
VALSQHPAALGTVAVKYQDMIAALPEATHEAIVGVGKQWSGARALEALLTVAGELRG
PPLQLDTGQLLKIAKRGGVTAVEAVHAWRNALTGAPLNLTPEQVVAIASNNGGKQAL
ETVQRLLPVLCQAHGLTPEQVVAIASNIGGKQALETVQRLLPVLCQAHGLTPDQVVA
IASNNGGKQALETVQRLLPVLCQAHGLTPEQVVAIASNGGGKQALETVQRLLPVLCQ
AHGLTPEQVVAIASNGGGKQALETVQRLLPVLCQAHGLTPEQVVAIASNGGGKQALE
TVQRLLPVLCQAHGLTPEQVVAIASNNGGKQALETVQRLLPVLCQAHGLTPEQVVAI
ASNIGGKQALETVQRLLPVLCQAHGLTPDQVVAIASNGGGKQALETVQRLLPVLCQA
HGLTPEQVVAIASNNGGKQALETVQRLLPVLCQAHGLTPEQVVAIASHDGGKQALET
VQRLLPVLCQAHGLTPAQVVAIASNGGGKQALETVQRLLPVLCQAHGLTPEQVVAIA
SHDGGKQALETVQRLLPVLCQAHGLTPAQVVAIASNIGGKQALETVQRLLPVLCQAH
GLTPDQVVAIASHDGGKQALETVQRLLPVLCQAHGLTPAQVVAIASHDGGKQALETV
QRLLPVLCQAHGLTPAQVVAIASHDGGKQALETVQRLLPVLCQAHGLTPAQVVAIAS

NGGGRPALESIVAQLSRPDPALAALTNDHLVALACLGGRPALDAVKKGLG*GS*GSYAL
GPYQISAPQLPAYNGQTVGTFYYVNDAGGLESKVFSSGG*SGGS*TNLSDIIEKETGKQ
LVIQESILMLPEEVEEVIGNKPESDILVHTAYDESTDENVMLLTSDAPEYKPWALVI
QDSNGENKIKML

Human G3733A Left TALE-G1333C

MLGFVGRVAAAPASGALRRLTPSASLPPAQLLLRAAPTAVHPVRDYAAQTSESGGGG
SPG*AAA*DYKDDDDK*GS*VDLRTLGYSQQQQEKIKPKVRSTVAQHHEALVGHGFTHAHI
VALSQHPAALGTVAVKYQDMIAALPEATHEAIVGVGKQWSGARALEALLTVAGELRG
PPLQLDTGQLLKIAKRGGVTAVEAVHAWRNALTGAPLNLTPEQVVAIASNIGGKQAL
ETVQRLLPVLCQAHGLTPDQVVAIASNNGGKQALETVQRLLPVLCQAHGLTPEQVVA
IASHDGGKQALETVQRLLPVLCQAHGLTPAQVVAIASHDGGKQALETVQRLLPVLCQ
AHGLTPAQVVAIASHDGGKQALETVQRLLPVLCQAHGLTPAQVVAIASNIGGKQALE
TVQRLLPVLCQAHGLTPDQVVAIASNIGGKQALETVQRLLPVLCQAHGLTPDQVVAI
ASNIGGKQALETVQRLLPVLCQAHGLTPDQVVAIASHDGGKQALETVQRLLPVLCQA
HGLTPAQVVAIASNIGGKQALETVQRLLPVLCQAHGLTPDQVVAIASNIGGKQALET
VQRLLPVLCQAHGLTPDQVVAIASNGGGKQALETVQRLLPVLCQAHGLTPEQVVAIA
SHDGGKQALETVQRLLPVLCQAHGLTPAQVVAIASNGGGRPALESIVAQLSRPDPAL
AALTNDHLVALACLGGRPALDAVKKGLG*GS*PTPYPNYANAGHVEGQSALFMRDNGIS
EGLVFHNNPEGTCGFCVNMTETLLPENAKMTVVPPEGAIPVKRGATGETKVFTGNSN
SPKSPTKGGC*SGGS*TNLSDIIEKETGKQLVIQESILMLPEEVEEVIGNKPESDILVH
TAYDESTDENVMLLTSDAPEYKPWALVIQDSNGENKIKML

Human G3733A Left TALE-G1397C

MLGFVGRVAAAPASGALRRLTPSASLPPAQLLLRAAPTAVHPVRDYAAQTSESGGGG
SPG*AAA*DYKDDDDK*GS*VDLRTLGYSQQQQEKIKPKVRSTVAQHHEALVGHGFTHAHI
VALSQHPAALGTVAVKYQDMIAALPEATHEAIVGVGKQWSGARALEALLTVAGELRG
PPLQLDTGQLLKIAKRGGVTAVEAVHAWRNALTGAPLNLTPEQVVAIASNIGGKQAL
ETVQRLLPVLCQAHGLTPDQVVAIASNNGGKQALETVQRLLPVLCQAHGLTPEQVVA
IASHDGGKQALETVQRLLPVLCQAHGLTPAQVVAIASHDGGKQALETVQRLLPVLCQ
AHGLTPAQVVAIASHDGGKQALETVQRLLPVLCQAHGLTPAQVVAIASNIGGKQALE
TVQRLLPVLCQAHGLTPDQVVAIASNIGGKQALETVQRLLPVLCQAHGLTPDQVVAI
ASNIGGKQALETVQRLLPVLCQAHGLTPDQVVAIASHDGGKQALETVQRLLPVLCQA
HGLTPAQVVAIASNIGGKQALETVQRLLPVLCQAHGLTPDQVVAIASNIGGKQALET
VQRLLPVLCQAHGLTPDQVVAIASNGGGKQALETVQRLLPVLCQAHGLTPEQVVAIA
SHDGGKQALETVQRLLPVLCQAHGLTPAQVVAIASNGGGRPALESIVAQLSRPDPAL
AALTNDHLVALACLGGRPALDAVKKGLG*GS*AIPVKRGATGETKVFTGNSNSPKSPTK
GGC*SGGS*TNLSDIIEKETGKQLVIQESILMLPEEVEEVIGNKPESDILVHTAYDEST
DENVMLLTSDAPEYKPWALVIQDSNGENKIKML

Human G3733A Left TALE-G1333N

MLGFVGRVAAAPASGALRRLTPSASLPPAQLLLRAAPTAVHPVRDYAAQTSESGGGG
SPG*AAA*DYKDDDDK*GS*VDLRTLGYSQQQQEKIKPKVRSTVAQHHEALVGHGFTHAHI
VALSQHPAALGTVAVKYQDMIAALPEATHEAIVGVGKQWSGARALEALLTVAGELRG

PPLQLDTGQLLKIAKRGGVTAVEAVHAWRNALTGAPLNLTPEQVVAIASNIGGKQAL
ETVQRLLPVLCQAHGLTPDQVVAIASNNGGKQALETVQRLLPVLCQAHGLTPEQVVA
IASHDGGKQALETVQRLLPVLCQAHGLTPAQVVAIASHDGGKQALETVQRLLPVLCQ
AHGLTPAQVVAIASHDGGKQALETVQRLLPVLCQAHGLTPAQVVAIASNIGGKQALE
TVQRLLPVLCQAHGLTPDQVVAIASNIGGKQALETVQRLLPVLCQAHGLTPDQVVAI
ASNIGGKQALETVQRLLPVLCQAHGLTPDQVVAIASHDGGKQALETVQRLLPVLCQA
HGLTPAQVVAIASNIGGKQALETVQRLLPVLCQAHGLTPDQVVAIASNIGGKQALET
VQRLLPVLCQAHGLTPDQVVAIASNGGGKQALETVQRLLPVLCQAHGLTPEQVVAIA
SHDGGKQALETVQRLLPVLCQAHGLTPAQVVAIASNGGGRPALESIVAQLSRPDPAL
AALTNDHLVALACLGGRPALDAVKKGLG*GS*GSYALGPYQISAPQLPAYNGQTVGTFY
YVNDAGGLESKVFSSGG*SGGS*TNLSDIIEKETGKQLVIQESILMLPEEVEEVIGNKP
ESDILVHTAYDESTDENVMLLTSDAPEYKPWALVIQDSNGENKIKML

Human G3733A Left TALE-G1397N

MLGFVGRVAAAPASGALRRLTPSASLPPAQLLLRAAPTAVHPVRDYAAQTSESGGGG
SPG*AAA*DYKDDDDK*GS*VDLRTLGYSQQQQEKIKPKVRSTVAQHHEALVGHGFTHAHI
VALSQHPAALGTVAVKYQDMIAALPEATHEAIVGVGKQWSGARALEALLTVAGELRG
PPLQLDTGQLLKIAKRGGVTAVEAVHAWRNALTGAPLNLTPEQVVAIASNIGGKQAL
ETVQRLLPVLCQAHGLTPDQVVAIASNNGGKQALETVQRLLPVLCQAHGLTPEQVVA
IASHDGGKQALETVQRLLPVLCQAHGLTPAQVVAIASHDGGKQALETVQRLLPVLCQ
AHGLTPAQVVAIASHDGGKQALETVQRLLPVLCQAHGLTPAQVVAIASNIGGKQALE
TVQRLLPVLCQAHGLTPDQVVAIASNIGGKQALETVQRLLPVLCQAHGLTPDQVVAI
ASNIGGKQALETVQRLLPVLCQAHGLTPDQVVAIASHDGGKQALETVQRLLPVLCQA
HGLTPAQVVAIASNIGGKQALETVQRLLPVLCQAHGLTPDQVVAIASNIGGKQALET
VQRLLPVLCQAHGLTPDQVVAIASNGGGKQALETVQRLLPVLCQAHGLTPEQVVAIA
SHDGGKQALETVQRLLPVLCQAHGLTPAQVVAIASNGGGRPALESIVAQLSRPDPAL
AALTNDHLVALACLGGRPALDAVKKGLG*GS*GSYALGPYQISAPQLPAYNGQTVGTFY
YVNDAGGLESKVFSSGGPTPYPNYANAGHVEGQSALFMRDNGISEGLVFHNNPEGTC
GFCVNMTETLLPENAKMTVVPPEG*SGGS*TNLSDIIEKETGKQLVIQESILMLPEEVE
EVIGNKPESDILVHTAYDESTDENVMLLTSDAPEYKPWALVIQDSNGENKIKML

Human G3733A Right TALE-G1333C

MLGFVGRVAAAPASGALRRLTPSASLPPAQLLLRAAPTAVHPVRDYAAQTSESGGGG
SPG*AAA*DYKDDDDK*GS*VDLRTLGYSQQQQEKIKPKVRSTVAQHHEALVGHGFTHAHI
VALSQHPAALGTVAVKYQDMIAALPEATHEAIVGVGKQWSGARALEALLTVAGELRG
PPLQLDTGQLLKIAKRGGVTAVEAVHAWRNALTGAPLNLTPEQVVAIASNIGGKQAL
ETVQRLLPVLCQAHGLTPDQVVAIASNNGGKQALETVQRLLPVLCQAHGLTPEQVVA
IASNGGGKQALETVQRLLPVLCQAHGLTPEQVVAIASNIGGKQALETVQRLLPVLCQ
AHGLTPDQVVAIASNNGGKQALETVQRLLPVLCQAHGLTPEQVVAIASNIGGKQALE
TVQRLLPVLCQAHGLTPDQVVAIASNIGGKQALETVQRLLPVLCQAHGLTPDQVVAI
ASNGGGKQALETVQRLLPVLCQAHGLTPEQVVAIASNNGGKQALETVQRLLPVLCQA
HGLTPEQVVAIASNIGGKQALETVQRLLPVLCQAHGLTPDQVVAIASNGGGKQALET
VQRLLPVLCQAHGLTPEQVVAIASNNGGKQALETVQRLLPVLCQAHGLTPEQVVAIA
SNNGGKQALETVQRLLPVLCQAHGLTPEQVVAIASHDGGKQALETVQRLLPVLCQAH

GLTPAQVVAIASNGGGRPALESIVAQLSRPDPALAALTNDHLVALACLGGRPALDAVKKGLG*GS*PTPYPNYANAGHVEGQSALFMRDNGISEGLVFHNNPEGTCGFCVNMTETLLPENAKMTVVPPEGAIPVKRGATGETKVFTGNSNSPKSPTKGGC*SGGS*TNLSDIIEKETGKQLVIQESILMLPEEVEEVIGNKPESDILVHTAYDESTDENVMLLTSDAPEYKPWALVIQDSNGENKIKML

Human G3733A Right TALE-G1397C

MLGFVGRVAAAPASGALRRLTPSASLPPAQLLLRAAPTAVHPVRDYAAQTSESGGGGSPG*AAA*DYKDDDDK*GS*VDLRTLGYSQQQQEKIKPKVRSTVAQHHEALVGHGFTHAHIVALSQHPAALGTVAVKYQDMIAALPEATHEAIVGVGKQWSGARALEALLTVAGELRGPPLQLDTGQLLKIAKRGGVTAVEAVHAWRNALTGAPLNLTPEQVVAIASNIGGKQALETVQRLLPVLCQAHGLTPDQVVAIASNNGGKQALETVQRLLPVLCQAHGLTPEQVVAIASNGGGKQALETVQRLLPVLCQAHGLTPEQVVAIASNIGGKQALETVQRLLPVLCQAHGLTPDQVVAIASNNGGKQALETVQRLLPVLCQAHGLTPEQVVAIASNIGGKQALETVQRLLPVLCQAHGLTPDQVVAIASNIGGKQALETVQRLLPVLCQAHGLTPDQVVAIASNGGGKQALETVQRLLPVLCQAHGLTPEQVVAIASNNGGKQALETVQRLLPVLCQAHGLTPEQVVAIASNIGGKQALETVQRLLPVLCQAHGLTPDQVVAIASNGGGKQALETVQRLLPVLCQAHGLTPEQVVAIASNNGGKQALETVQRLLPVLCQAHGLTPEQVVAIASNNGGKQALETVQRLLPVLCQAHGLTPEQVVAIASHDGGKQALETVQRLLPVLCQAHGLTPAQVVAIASNGGGRPALESIVAQLSRPDPALAALTNDHLVALACLGGRPALDAVKKGLG*GS*AIPVKRGATGETKVFTGNSNSPKSPTKGGC*SGGS*TNLSDIIEKETGKQLVIQESILMLPEEVEEVIGNKPESDILVHTAYDESTDENVMLLTSDAPEYKPWALVIQDSNGENKIKML

Human G3733A Right TALE-G1333N

MLGFVGRVAAAPASGALRRLTPSASLPPAQLLLRAAPTAVHPVRDYAAQTSESGGGGSPG*AAA*DYKDDDDK*GS*VDLRTLGYSQQQQEKIKPKVRSTVAQHHEALVGHGFTHAHIVALSQHPAALGTVAVKYQDMIAALPEATHEAIVGVGKQWSGARALEALLTVAGELRGPPLQLDTGQLLKIAKRGGVTAVEAVHAWRNALTGAPLNLTPEQVVAIASNIGGKQALETVQRLLPVLCQAHGLTPDQVVAIASNNGGKQALETVQRLLPVLCQAHGLTPEQVVAIASNGGGKQALETVQRLLPVLCQAHGLTPEQVVAIASNIGGKQALETVQRLLPVLCQAHGLTPDQVVAIASNNGGKQALETVQRLLPVLCQAHGLTPEQVVAIASNIGGKQALETVQRLLPVLCQAHGLTPDQVVAIASNIGGKQALETVQRLLPVLCQAHGLTPDQVVAIASNGGGKQALETVQRLLPVLCQAHGLTPEQVVAIASNNGGKQALETVQRLLPVLCQAHGLTPEQVVAIASNIGGKQALETVQRLLPVLCQAHGLTPDQVVAIASNGGGKQALETVQRLLPVLCQAHGLTPEQVVAIASNNGGKQALETVQRLLPVLCQAHGLTPEQVVAIASNNGGKQALETVQRLLPVLCQAHGLTPEQVVAIASHDGGKQALETVQRLLPVLCQAHGLTPAQVVAIASNGGGRPALESIVAQLSRPDPALAALTNDHLVALACLGGRPALDAVKKGLG*GS*GSYALGPYQISAPQLPAYNGQTVGTFYYVNDAGGLESKVFSSGG*SGGS*TNLSDIIEKETGKQLVIQESILMLPEEVEEVIGNKPESDILVHTAYDESTDENVMLLTSDAPEYKPWALVIQDSNGENKIKML

Human G3733A Right TALE-G1397N

MLGFVGRVAAAPASGALRRLTPSASLPPAQLLLRAAPTAVHPVRDYAAQTSESGGGG
SPG*AAA*DYKDDDDK*GS*VDLRTLGYSQQQQEKIKPKVRSTVAQHHEALVGHGFTHAHI
VALSQHPAALGTVAVKYQDMIAALPEATHEAIVGVGKQWSGARALEALLTVAGELRG
PPLQLDTGQLLKIAKRGGVTAVEAVHAWRNALTGAPLNLTPEQVVAIASNIGGKQAL
ETVQRLLPVLCQAHGLTPDQVVAIASNNGGKQALETVQRLLPVLCQAHGLTPEQVVA
IASNGGGKQALETVQRLLPVLCQAHGLTPEQVVAIASNIGGKQALETVQRLLPVLCQ
AHGLTPDQVVAIASNNGGKQALETVQRLLPVLCQAHGLTPEQVVAIASNIGGKQALE
TVQRLLPVLCQAHGLTPDQVVAIASNIGGKQALETVQRLLPVLCQAHGLTPDQVVAI
ASNGGGKQALETVQRLLPVLCQAHGLTPEQVVAIASNNGGKQALETVQRLLPVLCQA
HGLTPEQVVAIASNIGGKQALETVQRLLPVLCQAHGLTPDQVVAIASNGGGKQALET
VQRLLPVLCQAHGLTPEQVVAIASNNGGKQALETVQRLLPVLCQAHGLTPEQVVAIA
SNNGGKQALETVQRLLPVLCQAHGLTPEQVVAIASHDGGKQALETVQRLLPVLCQAH
GLTPAQVVAIASNGGGRPALESIVAQLSRPDPALAALTNDHLVALACLGGRPALDAV
KKGLG*GS*GSYALGPYQISAPQLPAYNGQTVGTFYYVNDAGGLESKVFSSGGPTPYPN
YANAGHVEGQSALFMRDNGISEGLVFHNNPEGTCGFCVNMTETLLPENAKMTVVPPE
G*SGGS*TNLSDIIEKETGKQLVIQESILMLPEEVEEVIGNKPESDILVHTAYDESTDE
NVMLLTSDAPEYKPWALVIQDSNGENKIKML

Human G8363A Left TALE

LTPEQVVAIASNIGGKQALETVQRLLPVLCQAHGLTPDQVVAIASNIGGKQALETVQ
RLLPVLCQAHGLTPDQVVAIASNNGGKQALETVQRLLPVLCQAHGLTPEQVVAIASN
IGGKQALETVQRLLPVLCQAHGLTPDQVVAIASNNGGKQALETVQRLLPVLCQAHGL
TPEQVVAIASNIGGKQALETVQRLLPVLCQAHGLTPDQVVAIASNIGGKQALETVQR
LLPVLCQAHGLTPDQVVAIASHDGGKQALETVQRLLPVLCQAHGLTPAQVVAIASHD
GGKQALETVQRLLPVLCQAHGLTPAQVVAIASNIGGKQALETVQRLLPVLCQAHGLT
PDQVVAIASNIGGKQALETVQRLLPVLCQAHGLTPDQVVAIASHDGGKQALETVQRL
LPVLCQAHGLTPAQVVAIASNIGGKQALETVQRLLPVLCQAHGLTPDQVVAIASHDG
GKQALETVQRLLPVLCQAHGLTPAQVVAIASHDGGKQALETVQRLLPVLCQAHGLTP
AQVVAIASNGGGKQALETVQRLLPVLCQAHGLTPEQVVAIASHDGGKQALETVQRLL
PVLCQAHGLTPAQVVAIASNGGGRPALE

Human G8363A Right TALE

LTPEQVVAIASNIGGKQALETVQRLLPVLCQAHGLTPDQVVAIASHDGGKQALETVQ
RLLPVLCQAHGLTPAQVVAIASNNGGKQALETVQRLLPVLCQAHGLTPEQVVAIASN
NGGKQALETVQRLLPVLCQAHGLTPEQVVAIASNGGGKQALETVQRLLPVLCQAHGL
TPEQVVAIASNIGGKQALETVQRLLPVLCQAHGLTPDQVVAIASNNGGKQALETVQR
LLPVLCQAHGLTPEQVVAIASNGGGKQALETVQRLLPVLCQAHGLTPEQVVAIASNI
GGKQALETVQRLLPVLCQAHGLTPDQVVAIASNGGGKQALETVQRLLPVLCQAHGLT
PEQVVAIASNGGGKQALETVQRLLPVLCQAHGLTPEQVVAIASNGGGKQALETVQRL
LPVLCQAHGLTPEQVVAIASNIGGKQALETVQRLLPVLCQAHGLTPDQVVAIASNNG
GKQALETVQRLLPVLCQAHGLTPEQVVAIASNGGGKQALETVQRLLPVLCQAHGLTP
EQVVAIASNGGGRPALE

Human G13513A Left TALE

LTPEQVVAIASHDGGKQALETVQRLLPVLCQAHGLTPAQVVAIASHDGGKQALETVQ
RLLPVLCQAHGLTPAQVVAIASNGGGKQALETVQRLLPVLCQAHGLTPEQVVAIASH
DGGKQALETVQRLLPVLCQAHGLTPAQVVAIASNIGGKQALETVQRLLPVLCQAHGL
TPDQVVAIASHDGGKQALETVQRLLPVLCQAHGLTPAQVVAIASNIGGKQALETVQR
LLPVLCQAHGLTPDQVVAIASNNGGKQALETVQRLLPVLCQAHGLTPEQVVAIASNN
GGKQALETVQRLLPVLCQAHGLTPEQVVAIASNGGGKQALETVQRLLPVLCQAHGLT
PEQVVAIASNGGGKQALETVQRLLPVLCQAHGLTPEQVVAIASNGGGKQALETVQRL
LPVLCQAHGLTPEQVVAIASHDGGKQALETVQRLLPVLCQAHGLTPAQVVAIASNGG
GKQALETVQRLLPVLCQAHGLTPEQVVAIASNIGGKQALETVQRLLPVLCQAHGLTP
DQVVAIASHDGGKQALETVQRLLPVLCQAHGLTPAQVVAIASNGGGRPALE

Human G13513A Right TALE

LTPEQVVAIASNNGGKQALETVQRLLPVLCQAHGLTPEQVVAIASNGGGKQALETVQ
RLLPVLCQAHGLTPEQVVAIASNGGGKQALETVQRLLPVLCQAHGLTPEQVVAIASN
GGGKQALETVQRLLPVLCQAHGLTPEQVVAIASNNGGKQALETVQRLLPVLCQAHGL
TPEQVVAIASHDGGKQALETVQRLLPVLCQAHGLTPAQVVAIASNNGGKQALETVQR
LLPVLCQAHGLTPEQVVAIASNNGGKQALETVQRLLPVLCQAHGLTPEQVVAIASNG
GGKQALETVQRLLPVLCQAHGLTPEQVVAIASNGGGKQALETVQRLLPVLCQAHGLT
PEQVVAIASNGGGKQALETVQRLLPVLCQAHGLTPEQVVAIASHDGGKQALETVQRL
LPVLCQAHGLTPAQVVAIASNNGGKQALETVQRLLPVLCQAHGLTPEQVVAIASNIG
GKQALETVQRLLPVLCQAHGLTPDQVVAIASNGGGRPALE
